# Outcomes associated with antithrombotic strategies in heart failure with reduced ejection fraction and sinus rhythm following acute ischemic stroke

**DOI:** 10.3389/fneur.2022.1041806

**Published:** 2022-12-15

**Authors:** Parth Patel, Justin Tiongson, Austin Chen, Taylor Siegal, Solomon Oak, Akhil Golla, Scott Kamen, Jesse M. Thon, Nicholas Vigilante, Ameena Rana, Taryn Hester, James E. Siegler

**Affiliations:** ^1^Cooper Medical School of Rowan University, Camden, NJ, United States; ^2^Cooper Neurological Institute, Cooper University Hospital, Camden, NJ, United States; ^3^Department of Neurology, Sinai Beth Israel Hospital, New York, NY, United States

**Keywords:** anticoagulant, antiplatelet, embolism, heart failure, stroke

## Abstract

**Purpose:**

Insufficient data exist regarding the benefit of long-term antiplatelet vs. anticoagulant therapy in the prevention of recurrent ischemic stroke in patients with ischemic stroke and heart failure with reduced ejection fraction (HFrEF). Therefore, this study aimed to compare longitudinal outcomes associated with antiplatelet vs. anticoagulant use in a cohort of patients with stroke and with an ejection fraction of ≤40%.

**Methods:**

We retrospectively analyzed single-center registry data (2015–2021) of patients with ischemic stroke, HFrEF, and sinus rhythm. Time to the primary outcome of recurrent ischemic stroke, major bleeding, or death was assessed using the adjusted Cox proportional hazards model and was compared between patients treated using anticoagulation (±antiplatelet) vs. antiplatelet therapy alone after propensity score matching using an intention-to-treat (ITT) approach, with adjustment for residual measurable confounders. Sensitivity analyses included the multivariable Cox proportional hazards model using ITT and as-treated approaches without propensity score matching.

**Results:**

Of 2,974 screened patients, 217 were included in the secondary analyses, with 130 patients matched according to the propensity score for receiving anticoagulation treatment for the primary analysis, spanning 143 patient-years of follow-up. After propensity score matching, there was no significant association between anticoagulation and the primary outcome [hazard ratio (HR) 1.10, 95% confidence interval (CI): 0.56–2.17]. Non-White race (HR 2.26, 95% CI: 1.16–4.41) and the presence of intracranial occlusion (HR 2.86, 95% CI: 1.40–5.83) were independently associated with the primary outcome, while hypertension was inversely associated (HR 0.42, 95% CI: 0.21–0.84). There remained no significant association between anticoagulation and the primary outcome in sensitivity analyses.

**Conclusion:**

In HFrEF patients with an acute stroke, there was no difference in outcomes of antithrombotic strategies. While this study was limited by non-randomized treatment allocation, the results support future trials of stroke patients with HFrEF which may randomize patients to anticoagulation or antiplatelet.

## Introduction

Heart failure with reduced ejection fraction (HFrEF) and sinus rhythm are seen in a minority of patients with embolic stroke of undetermined source (ESUS) (1), with an estimated U.S. prevalence of 6.5 million, which is expected to increase in the coming years (2). While it is likely that strokes in the setting of HFrEF are embolic in nature, only one *post hoc* analysis of a randomized clinical trial has shown the superiority of anticoagulation over antiplatelet therapy for primary prevention of cerebral embolism (3). The advantage of anticoagulation over antiplatelet is largely counterbalanced by the risk of major bleeding among published randomized controlled trials on primary prevention (4). Furthermore, no randomized clinical trials have tested the superiority of anticoagulation for *secondary* stroke prevention following ischemic stroke in this population. For this reason, there is significant variation in prescription patterns in antithrombotic therapy for patients with HFrEF and acute ischemic stroke (5). Given the small but significant benefit of anticoagulation with full-dose rivaroxaban over aspirin in HFrEF for primary prevention (3, 6), there is likely an equivalent—if not greater—advantage of anticoagulation in patients with prior cerebral infarction.

To evaluate whether anticoagulation is associated with a clinical benefit over antiplatelet therapy in patients with HFrEF, we evaluated longitudinal outcomes in a cohort of survivors with stroke and HFrEF in sinus rhythm.

## Methods

Data will be made available to any qualified investigator upon reasonable request.

### Study design and participants

Patients with consecutive acute ischemic stroke were screened from a consolidated retrospective (01/2015–08/2019) and prospective (09/2019–07/2021) registry for inclusion. Patients were eligible for the inclusion in this analysis if they underwent transthoracic or transesophageal echocardiography during stroke admission (or in the preceding 3 months) with a documented ejection fraction of 40% or less, if they survived more than 7 days from the index stroke event, and if they were treated with anticoagulation (vitamin K antagonist, direct oral anticoagulant, or heparin product) or antiplatelet therapy or a combination of these. A left ventricular ejection fraction of ≤40% was used based on recent trial data suggesting anticoagulation may be associated with a reduction in index stroke events in patients with this degree of ventricular dysfunction, as compared with aspirin (6, 7). Patients were excluded if they were not treated with any antithrombotic agent due to risk of bleeding or active bleeding, were discharged to hospice within 7 days of the index event, had another indication for anticoagulation therapy (e.g., concomitant atrial fibrillation), or were thought to have transient ventricular dysfunction on echocardiography (e.g., stress-related ventricular dysfunction).

### Data collection

The left atrial (LA) diameter was measured by two-dimensional echocardiography in the apical two- and four-chamber views during the end of the systole by licensed echocardiographic technicians in accordance with guidelines established by the American Society of Echocardiography (8). LAE was reported in two ways: (1) quantitatively (in cm) for 254 of the included patients (*n* = 22 missing) and qualitatively (categorized as normal, mild, moderate, and severe enlargement) for 274 of the included patients (*n* = 2 missing). Among them, two patients had poor visualization of the left atrium on echocardiography, and neither qualitative nor quantitative estimation could be abstracted. The severity of LAE was defined in accordance with recommendations by the American Society of Echocardiography and the European Association of Echocardiography as mild (4.1–4.6 cm in male patients or 3.9–4.2 cm in female patients), moderate (4.7–5.1 cm in male patients or 4.3–4.6 cm in female patients), or severe (≥5.2 cm in male patients or ≥4.7 cm in female patients) (9). When both quantitative and qualitative left atrial measurements were provided, the quantitative measurement was utilized to determine the LAE severity category.

### Statistical analyses

Patients were grouped according to antithrombotic treatment as those treated with antiplatelet alone (aspirin, clopidogrel, prasugrel, ticagrelor, and/or cilostazol) and those treated with anticoagulation (warfarin, apixaban, dabigatran, rivaroxaban, heparin/low-molecular weight heparin, and fondaparinux) with or without antiplatelet therapy. Patients in the antiplatelet therapy group could have been treated with monotherapy or combination antiplatelet therapy, while patients in the anticoagulation group could have been treated with anticoagulation ± any antiplatelet agent(s). Continuous variables are reported as medians with interquartile range and compared using the Kruskal–Wallis equality of populations rank test. Categorical variables are reported as proportions and compared using the χ^2^ test or Fisher's exact test when contingency table cell counts were <5, as appropriate.

Propensity score matching (PSM) was used to balance treatment groups according to treatment with anticoagulation ± antiplatelet vs. antiplatelet therapy (10). PS estimates were generated for each patient according to the probability of receiving anticoagulation at discharge, conditional on the following clinical characteristics: age, sex, Hispanic ethnicity, non-White race, history of hypertension, diabetes mellitus, dyslipidemia, tobacco use, heart failure, left ventricular ejection fraction, and the presence of an intracranial occlusion on non-invasive imaging. Patients were matched 1:1 according to the propensity for receiving anticoagulation using the nearest neighbor method with a caliper of 0.25, without replacement. Standardized differences (SD) are reported by convention, with differences of ±0.20 indicating a significant imbalance.

The primary outcome was a composite of recurrent ischemic stroke, major bleeding, or death at follow-up. Major bleeding was defined by the International Society of Thrombosis and Hemostasis (11). Differences in time to composite event were compared between the PSM treatment groups using unadjusted Cox proportional hazards regression analysis and are further reported in annualized event rates with corresponding 95% confidence intervals (95% CI). Due to the residual imbalance of measured confounders, the Cox proportional hazards models were adjusted for characteristics that remained associated with the primary (composite) outcome to *p* < 0.2 in unadjusted Cox regression (non-White race, history of hypertension, and presence of an intracranial occlusion). All models were generated using the intention-to-treat (ITT) principle, according to antithrombotic recommendations at the time of discharge following the index stroke event (or antithrombotic recommendation 7 days after the event, for patients, still hospitalized). The proportional hazards assumption was tested for each model by visualization of the survival curves and log-log survival plots, and test of Schoenfeld residuals. Due to the high incidence rate of death, we fitted a competing risks regression model in the PSM cohort, with adjustment for non-White race, history of hypertension, and intracranial occlusion. In this competing risks regression, the censored event was a composite of recurrent stroke or major bleeding, with death due to any cause as a competing risk (which could have impeded later outcomes of stroke or major bleeding). The subhazard ratio with corresponding 95% CI was calculated to estimate the association between antithrombotic type and stroke/major bleeding.

A secondary analysis was performed using the cohort of patients who met study inclusion, irrespective of those matched by propensity score adjustment. In this analysis, patient outcomes were compared between treatment groups using a modified ITT principle (as in the primary PSM analysis). This secondary analysis assessed time to the composite event using the Cox proportional hazards model, with multivariable adjustment as described earlier. Survival curves were constructed using Kaplan–Meier estimates, with differences described as adjusted hazard ratios with corresponding 95% confidence intervals.

All tests were performed at the two-sided level, with an alpha set at 0.05. Missing data were minimal and not imputed. At the time of the last follow-up, one patient died but was classified as having a missing stroke outcome due to a lack of confirmatory imaging prior to death. Analyses were performed using STATA 15.0 (College Station, TX). Sample size estimates for the present study were derived from a *post hoc* analysis (12) of patients with ESUS and HFrEF, indicating a ~5% absolute risk reduction of stroke/embolism, myocardial infarction, or death with anticoagulation vs. antiplatelet therapy (4.9 vs. 9.5% annualized risk), with greater risk of stroke in patients with more severe left ventricular dysfunction and doubled risk of patients with very reduced ejection fraction (13). However, these trials included patients with minimal premorbid disability; therefore, we anticipated our rate of recurrent stroke and death to be greater in our population [which includes patients with pre-existing and ongoing disability (14)]. Assuming an absolute difference in the primary event rate of 20% between treatment groups (with 10% of patients on antiplatelet therapy anticipated to experience the primary outcome), a sample size of 184 (92 in each arm) would provide 80% power to detect a significant difference. For this reason, secondary analyses were conducted using multivariable regression in order to achieve greater statistical power. These results are reported in accordance with the Strengthening the Reporting of Observational Studies in Epidemiology guidelines.

In addition to the formal analyses conducted on this dataset, we performed a systematic literature review identifying abstracts of published randomized clinical trials (1980–2022) evaluating outcomes of anticoagulation vs. antiplatelet treatment for patients with heart failure and sinus rhythm. PubMed and Medline were searched for the keywords “heart failure”; and “anticoagulation,” “warfarin,” “apixaban,” “dabigatran,” “dabigatran,” “edoxaban,” “heparin”; and “randomized clinical trial” for randomized clinical trials or *post hoc* analyses of randomized trials. The references in those trials were also screened for potential trials that could be included in the systematic review. The results of this review are summarized in a narrative form.

## Results

### Intention-to-treat analysis according to PSM

**Figure 1:**
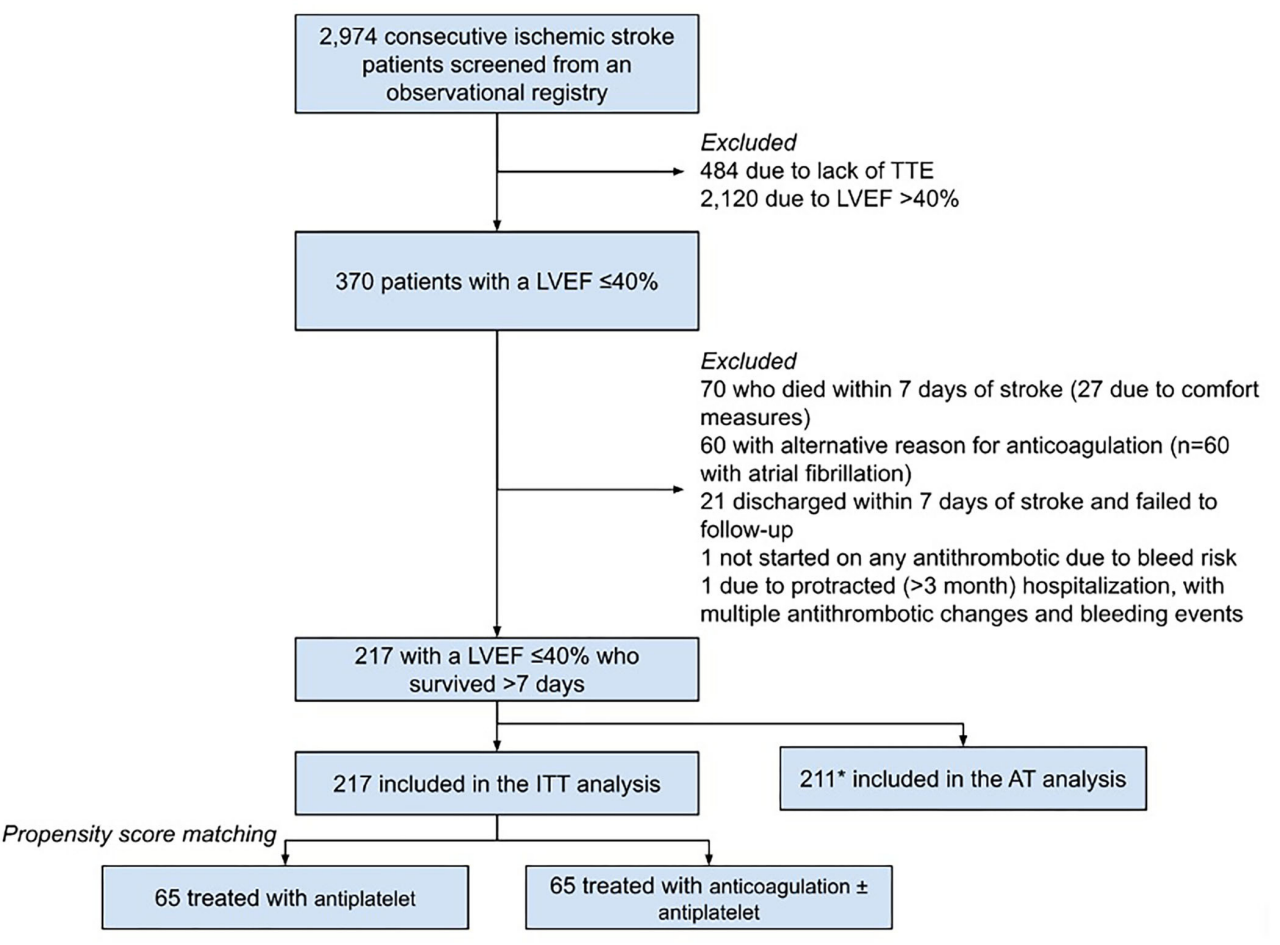
Inclusion flowchart. *Six patients from the ITT were not included in AT due to discontinuation of antithrombotics at last follow-up for reasons other than significant bleeding event (1 with unclear documentation, 5 with self-discontinuation/non-adherence). TTE, transthoracic echocardiogram; LVEF, left ventricular ejection fraction; ITT, intention-to-treat; AT, as-treated.

Of the 2,974 patients screened for inclusion, 217 had treatment data for the ITT analysis (cumulative follow-up of 223.9 patient-years), 130 of whom were propensity-matched (*n* = 65 in each arm, cumulative follow-up of 143.0 patient-years) without significant residual imbalance of baseline characteristics (Table 1; Figure 1). In the PSM cohort, the median time to follow-up was 184 (IQR 32–613) days, patients had a median age of 64 (IQR 55–71) years, and 33 (25.4%) were female, with a median CHA_2_DS_2_Vasc score of 4 (IQR 3–5). The median LVEF was 30% (IQR 20–35) with 36 patients (27.7%) having sex-adjusted moderate-to-severe left atrial enlargement on TTE and 29 (22.3%) having an intracranial occlusion. In the PSM anticoagulation group, 15 of the 65 patients (23.1%) were discharged on warfarin, and 12 (18.5%) were on warfarin at the time of the last follow-up; and in the non-PSM ITT cohort, 29 of 115 patients (25.2%) were discharged on warfarin, and 23 (20.0%) were on warfarin at the time of the last follow-up.

**Table 1:** Demographics according to intention-to-treat principle.

	**Unadjusted cohort**			**Propensity score matched cohort**		
	**Antiplatelet** **(*n* = 102)**	**Anticoagulation ±antiplatelet** **(*n* = 115)**	**Standardized difference**	**Antiplatelet** **(*n* = 65)**	**Anticoagulation ±antiplatelet** **(*n* = 65)**	**Standardized difference**
Age, median year (IQR)	63 (56–70)	69 (58–76)	−0.33	63 (56–71)	64 (55–72)	0.06
Female, no. (%)	25 (24.5%)	43 (37.4%)	−0.28	19 (29.2%)	14 (21.5%)	0.18
Race, no. (%)			0.09			0.17
White	49/98 (50.0%)	60/113 (53.1%)		37 (56.9%)	32 (49.2%)	
Black	35/98 (35.7%)	40/113 (35.4%)		20 (30.8%)	25 (38.5%)	
Other	14/98 (14.3%)	13/113 (11.5%)		8 (12.3%)	8 (12.3%)	
Hispanic, no. (%)	12/93 (12.9%)	11/113 (9.8%)	0.10	6 (9.2%)	7 (10.8%)	−0.05
Past medical history, no. (%)						
Hypertension	59 (57.8%)	45 (39.1%)	0.38	31 (47.7%)	29 (44.6%)	0.06
Diabetes mellitus	29 (28.4%)	21 (18.3%)	0.24	14 (21.5%)	14 (21.5%)	< 0.01
Dyslipidemia	29 (28.4%)	35 (30.4%)	−0.04	17 (26.2%)	17 (26.2%)	< 0.01
Congestive heart failure	18 (17.7%)	9 (7.8%)	0.30	5 (7.7%)	7 (10.8%)	−0.11
Prior stroke	26 (25.5%)	24 (20.9%)	0.11	17 (26.2%)	12 (18.5%)	0.18
Tobacco use	18 (17.7%)	17 (14.8%)	0.08	12 (18.5%)	10 (15.4%)	0.08
CHA_2_DS_2_-Vasc score, median (IQR)	4 (3–5)	4 (3–5)	0.02	4 (3–5)	3 (3–5)	0.09
Mod-severe atrial enlargement, no. (%)	35 (34.3%)	37 (32.2%)	0.05	16 (24.6%)	20 (30.8%)	−0.14
LVEF, median % (IQR)	30 (20–35)	30 (20–35)	< 0.01	30 (20–35)	30 (20–35)	0.04
Large vessel occlusion, no. (%)	32/99 (32.3%)	20/107 (18.7%)	0.32	13 (20.0%)	16 (24.6%)	−0.11
Antiplatelet(s) at discharge, no. (%)	102 (100.0%)	76 (66.1%)	1.01	65 (100.0%)	46 (70.8%)	0.90
Anticoagulant at discharge, no. (%)	0 (0.0%)	115 (100.0%)	n/a	0 (0.0%)	65 (100.0%)	n/a
Antiplatelet(s) at last follow-up, no. (%)	94/100 (94.0%)	71/111 (64.0%)	0.79	59 (93.7%)	43 (68.3%)	0.68
Anticoagulant at last follow-up, no. (%)	22/100 (22.0%)	99/111 (89.2%)	−1.83	14 (22.2%)	58 (92.1%)	−1.98

IQR, interquartile range; CHA2DS2-Vasc, congestive heart failure, hypertension, atrial fibrillation, diabetes, stroke, and peripheral vascular disease; LVEF, left ventricular ejection fraction. Standardized differences of ±0.20 indicate significant imbalance.

**Figure 2:**
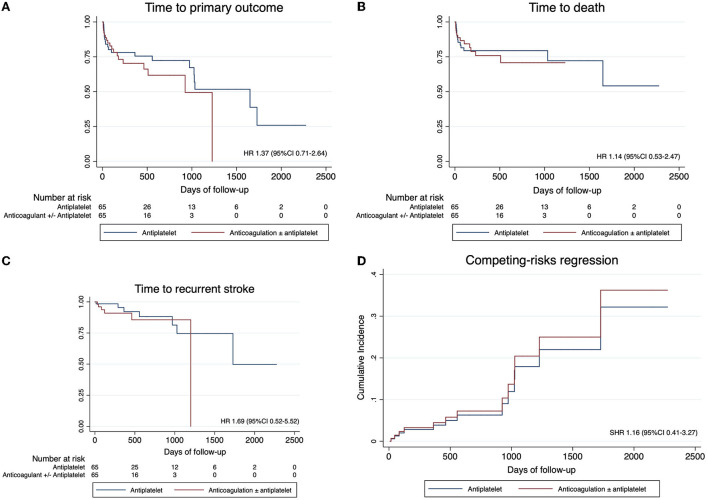
Survival estimates for the primary and secondary outcomes in the propensity score-matched cohort. Survival estimates for **(A)** the primary outcome of recurrent stroke, major bleeding event, or death; **(B)** recurrent stroke; and **(C)** death due to any cause. **(D)** The cumulative incidence function of stroke or major bleeding, with death considered as a competing risk. Solid blue lines indicate treatment with antiplatelet, while dashed red lines indicate treatment with anticoagulant ± antiplatelet. HR, hazard ratio; CI, confidence interval; SHR, subhazard ratio. Note that major bleeding events were too few in number; therefore, survival estimates are not shown.

There was a trend toward a longer duration of follow-up among patients treated with antiplatelet over anticoagulation than those treated with antiplatelet therapy [median 289 days (IQR 32–905) vs. 127 days (IQR 46–463), *p* = 0.08, SD = 0.46]. The annualized risk of the primary outcome was 28.0% (95% CI: 20.5–38.1%). The absolute event rates of the primary outcome of recurrent stroke, major bleeding, or death were not different between the anticoagulation vs. antiplatelet groups (29.2 vs. 32.3%, *p* = 0.70, SD = 0.06; Table 2), with no difference in the cumulative hazard between the two groups in the unadjusted Cox regression (HR 1.37, 95% CI: 0.71–2.64; Figure 2). With adjustment for residual confounders (non-White race, hypertension, and presence of an intracranial occlusion), there remained no association between anticoagulation and the primary outcome in the primary PSM model (HR 1.10, 95% CI: 0.56–2.17). Independent predictors of the primary outcome included intracranial occlusion at the index event (HR 2.86, 95% CI: 1.40–5.83) and non-White race (HR 2.26, 95% CI: 1.16–4.41), while hypertension was protective of the primary outcome (HR 0.42, 95% CI: 0.21–0.84). In the competing risks regression model, with death as a competing event for recurrent stroke or major bleeding, there was no association between anticoagulation and recurrent stroke or bleeding (subhazard ratio 1.16, 95% CI: 0.41–3.27; Figure 2D).

**Table 2:** Primary and secondary outcomes from the intention-to-treat analysis by PSM.

	**Propensity score matched groups**			**Unadjusted hazard ratio**	
	**Antiplatelet (*n* = 65)**	**Anticoagulation ±antiplatelet (*n* = 65)**	**Standardized difference**	**HR (95%CI)**	***p***
Follow-up duration, median days (IQR)	289 (32–905)	127 (46–463)	0.46	–	–
Primary outcome					
Recurrent stroke, major bleeding, or death	21 (32.3%)	19 (29.2%)	0.07	1.37 (0.71–2.64)	0.35
Secondary outcomes					
Recurrent stroke	7 (10.8%)	6 (9.2%)	0.05	1.69 (0.52–5.52)	0.39
Major bleeding	2 (3.1%)	1 (1.5%)	0.10	1.09 (0.09–12.91)	0.95
Death^†^	14 (21.5%)	13 (20.0%)	0.04	1.14 (0.53–2.47)	0.74

PSM, propensity score matching; HR, hazard ratio; CI, confidence interval; IQR, interquartile range. Standardized differences of ±0.20 indicate significant imbalance.

† Death rates were counted if major bleed or stroke contributed to patient death (n = 2 for antiplatelet group, n = 1 for anticoagulant group).

Among the secondary outcomes, the annualized risk of stroke recurrence in the entire cohort was 9.2% (95% CI: 5.4–15.9%), and death was 18.9% (95% CI: 12.9–27.5%). Anticoagulation was not associated with any significant difference in recurrent stroke risk (HR 1.69, 95% CI: 0.52–5.52) or death (HR 1.14, 95% CI: 0.53–2.47; Figure 2). Major bleeding events were infrequent (*n* = 3 out of 130 patients with PSM).

### Secondary multivariable analysis

In unadjusted Cox regression including the original cohort of 217 patients (*n* = 115 treated with anticoagulation), there remained no association between anticoagulation and the primary outcome (HR 1.39, 95% CI: 0.82 PSM 2.35), and no association following multivariable adjustment (HR 1.31, 95% CI: 0.75–2.30). In the multivariable model, independent predictors of the primary outcome included large-vessel occlusion at the index event (HR 1.97, 95% CI: 1.07–3.61) and non-White race (HR 1.88, 95% CI: 1.07–3.29), while hypertension was protective (HR 0.43, 95% CI: 0.19–0.98).

Among the secondary outcomes, the annualized risk of stroke recurrence in the entire cohort was 7.3% (95% CI: 4.5–12.0%), and death was 17.9% (95% CI: 13.1–24.4%). Anticoagulation was not associated with any significant difference in recurrent stroke risk in unadjusted (HR 1.69, 95% CI: 0.62–4.62) or adjusted regression (HR 1.74, 95% CI: 0.64–4.79). In this model, ejection fraction was the only additional covariate (*p* = 0.09 in univariate analysis), and it was non-significantly but inversely associated with stroke recurrence in the adjusted model (adjusted HR 0.95 per %, 95% CI: 0.90–1.01). In the sensitivity analysis comparing direct oral anticoagulants (*n* = 70) and low-molecular weight heparin (*n* = 16) against antiplatelet use, there remained no association between anticoagulation and a lower risk of recurrent stroke in unadjusted (HR 1.45, 95% CI: 0.49–4.26) or adjusted regression (HR 1.58, 95% CI: 0.0.53–4.73). The risk of death was not associated with anticoagulant use in unadjusted or adjusted regression; however, death was strongly associated with large-vessel occlusion at the time of index event (HR 2.34, 95% CI: 1.11–4.93), with non-White race trending toward significance (HR 1.69, 95% CI: 0.85–3.37), and female sex (HR 0.39, 95% CI: 0.16–0.95) and prior hypertension (HR 0.31, 95% CI: 0.12–0.79) being protective of the outcome of death. Major bleeding was not assessed in multivariable regression due to the low event rates (*n* = 4).

In the sensitivity analysis comparing direct oral anticoagulants (*n* = 70) and low-molecular weight heparin (*n* = 16) against antiplatelet use, there remained no association between anticoagulation and a lower risk of recurrent stroke in unadjusted (HR 1.45, 95% CI: 0.49–4.26) or adjusted regression (HR 1.58, 95% CI: 0.0.53–4.73). The risk of death was not associated with anticoagulant use in unadjusted or adjusted regression; however, death was strongly associated with large-vessel occlusion at the time of index event (HR 2.34, 95% CI: 1.11–4.93), with non-White race trending toward significance (HR 1.69, 95% CI: 0.85–3.37), and female sex (HR 0.39, 95% CI: 0.16–0.95) and prior hypertension (HR 0.31, 95% CI: 0.12–0.79) being protective of the outcome of death. Major bleeding was not assessed in multivariable regression due to the low event rates (*n* = 4).

## Discussion

In our single-center, longitudinal cohort study of patients with acute ischemic stroke and moderate-to-severe left ventricular dysfunction spanning 140 patient-years in the PSM analysis, we found no significant difference in the risk of recurrent stroke, major bleeding, or death according to treatment with anticoagulation or antiplatelet therapy. While the initial analysis sought to compare outcomes between patients treated with anticoagulation and those treated with antiplatelet agents, more than two-thirds of patients treated using anticoagulation were on concomitant antiplatelet medications. Therefore, we cannot conclude that there would be no differences in outcomes between patients treated with anticoagulation and those treated with antiplatelet monotherapy. Instead, anticoagulation when added to antiplatelet treatment is associated with no significant improvement in the rate of recurrent stroke, major bleeding, or death, as compared with antiplatelet alone. Notably, we observed a markedly high risk of the primary outcome of stroke, major bleeding, and death, which exceeds that reported in prior clinical trials of patients with HFrEF (1.2% annualized risk of stroke, 1.4% annualized risk of major bleeding, and 9.5% annualized risk of death) (4). The heightened risk of this composite outcome in our population likely reflects both a high degree of baseline illness among included patients seen at our center and the early time from index event to inclusion (7 days). Most events in the primary outcome were death, with an estimated 18.9 deaths per 100 person-years, a doubled risk as compared with previously published cohorts (15). We suspect most deaths were related to complications of acute stroke (e.g., disability-related events such as aspiration pneumonia) and may not have been influenced by antithrombotic treatment allocation. Furthermore, patients with stroke at our center often have a considerable pre-stroke disability, with one in six patients with stroke having a pre-stroke modified Rankin Scale score of 3 or 4, according to data from an overlapping cohort (16). These patients are at a high risk of death within 90 days (40–70%), many of whom did not have ventricular dysfunction (which should further increase the risk of death) (17). Therefore, the excess rate of early death may have blunted any difference seen in the primary composite outcome between the two treatment groups, and for this reason, we pursued subgroup analyses of each individual outcome.

The evidence for anticoagulation in patients with left ventricular dysfunction and sinus rhythm is conflicting, with a competing advantage of anticoagulation for stroke prevention and heightened risk of hemorrhage with anticoagulant use (Table 3). Largely, randomized trials published to date have evaluated the safety and efficacy of anticoagulation vs. antiplatelet therapy for the *primary* prevention of cardiovascular events, stroke/systemic embolism, and/or death. In general, there is an advantage of anticoagulation with warfarin or direct oral anticoagulants for the prevention of embolic events, which has been confirmed in a recent meta-analysis (4). In this meta-analysis, including data from five randomized clinical trials (*n* = 9,490 patients spanning 21,067 patient-years), anticoagulation was associated with a 1.3% absolute risk reduction and 40% lower odds of ischemic stroke (OR 0.60, 95% CI: 0.46–0.78), with a benefit observed in warfarin as well as direct oral anticoagulant use. However, this advantage came with two-fold greater odds of major hemorrhage (OR 1.92, 95% CI: 1.51–2.45). There was no reported survival advantage with anticoagulation over antiplatelet therapy, with the outcome of survival, rehospitalization for heart failure, or myocardial ischemia. The five trials referenced in this meta-analysis are presented in Table 3.

**Table 3:** Summary of published randomized clinical trials evaluating antithrombotic treatment in heart failure with reduced ejection fraction and sinus rhythm.

**First author/trial**	**Population**	**Treatment/exposure**	**Primary outcome**	**Result(s)**	**Comments**
Cleland/WASH (18)	279 patients with left ventricular dysfunction*	No antithrombotic vs. warfarin vs. aspirin for *primary* prevention	Composite: Death, non-fatal myocardial infarction, or non-fatal stroke	**No difference between warfarin and aspirin or placebo groups** 1.56 strokes/100 patient-years among all patients	Trial terminated following concerns that “no antithrombotic” arm would impede recruitment
Cokkinos/HELAS (19)	197 patients with LVEF < 35% and *either* IHD or DCM	Warfarin vs. aspirin for *secondary* prevention following myocardial infarction in IHD Warfarin vs. placebo for *primary* prevention in DCM	Composite: Non-fatal stroke, peripheral or pulmonary embolism, myocardial (re)infarction, re-hospitalization, exacerbation of heart failure, or death	**No difference between warfarin and aspirin or placebo groups** 15.7 events/100 patient-years for warfarin vs. 14.9 events/100 patient-years on aspirin with IHD 8.9 events/100 patient-years for warfarin vs. 14.8 events/100 patient-years on placebo with DCM 2.2 embolic events/100 patient-years among all patients	
Massie/WATCH (20)	1,587 patients with LVEF ≤ 35% and symptoms of heart failure	Open-label warfarin vs. randomized aspirin or clopidogrel for *primary* prevention	Composite: All-cause mortality, non-fatal MI, and non-fatal stroke	**No benefit of warfarin over aspirin** HR 0.98, 95%CI 0.86–1.12 **No benefit of warfarin over clopidogrel** HR 0.89, 95%CI 0.68–1.16 **No benefit of clopidogrel over aspirin** HR 1.08, 95%CI 0.83–1.40	Study was terminated prematurely due to slow enrollment (target enrollment 4,500 not met). Power to detect the original 20% difference in primary outcome dropped from 90 to 41% One-fifth of study participants discontinued original study drug, or crossed over to other treatment arm
Homma/WARCEF (21)	2,305 patients with LVEF ≤ 35%	Warfarin vs. aspirin for *primary* prevention	Composite: Ischemic stroke, intracerebral hemorrhage, or death	**No benefit of warfarin over aspirin** HR 0.93, 95%CI 0.79–1.10	Among patients who survived 4 or more years after enrollment, there was a time-dependent benefit of warfarin over aspirin in the prevention of the primary outcome
Zannad/COMMANDER HF (3, 6)	5,022 patients with CHF, LVEF ≤ 40% elevated plasma concentration of natriuretic peptides without atrial fibrillation	Rivaroxaban vs. aspirin for *primary* prevention	Efficacy: Composite outcome or death from any cause, myocardial infarction or stroke Safety: Fatal bleeding or bleeding into a critical space	**No benefit of rivaroxaban over aspirin for primary outcome** HR 0.94, 95%CI 0.84–1.05 **Rivaroxaban superior to aspirin for exploratory p*****ost hoc*** **outcome of systemic** **embolism** (3) HR 0.83, 95% CI 0.72–0.96	
Branch/COMPASS (22, 23)	Nested cohort/exploratory *post hoc analysis* of 4971 patients with coronary and/or peripheral artery disease and LVEF 31–40% from the COMPASS trial (*N* = 27,395)	Rivaroxaban with or without aspirin for *primary* prevention	Composite: Cardiovascular death, stroke, or myocardial infarction	**No benefit of rivaroxaban monotherapy over aspirin for primary outcome; benefit of low-dose rivaroxaban** **+** **aspirin was observed over aspirin** HR for half-dose rivaroxaban + aspirin vs. aspirin alone 0.68, 95%CI 0.53–0.86	The COMPASS trial included 3 arms: rivaroxaban 2.5 mg twice daily plus aspirin 100mg once daily vs. rivaroxaban 5 mg twice daily vs. aspirin 100 mg once daily. In this analysis, only the combination of half-dose rivaroxaban + aspirin was found to be associated with lower risk of the primary outcome vs. aspirin alone
Merkler/NAVIGATE-ESUS (12, 24)	Nested cohort/exploratory *post hoc analysis* of 502 patients with stroke and left ventricular dysfunction^†^ from the NAVIGATE-ESUS^†^ trial (*N* = 7,213)	Rivaroxaban vs. aspirin for *secondary* prevention of ischemic stroke	Composite: Recurrent stroke or systemic embolism	**No benefit of rivaroxaban over aspirin for the primary outcome in the principal cohort** **Benefit of rivaroxaban over aspirin was observed for primary outcome in patients with left ventricular dysfunction** HR 0.36; 95% CI, 0.14–0.93	Left ventricular ejection fraction not reported

* Left ventricular dysfunction from WASH was defined as increased left ventricular end-diastolic internal dimension (≥56 mm or ≥30 mm/m^2^ body surface area) combined with a fractional shortening of ≤ 28% or an echocardiographic ejection fraction ≤ 35%.

† NAVIGATE-ESUS classified left ventricular dysfunction when regional wall motion abnormalities were noted or there was moderate to severely impaired left ventricular contractility with or without regional wall motion abnormalities.

WASH, Warfarin/Aspirin Study in Heart failure; HELAS, Heart failure Long-term Antithrombotic Study; IHD, ischemic heart disease; DCM, dilated cardiomyopathy; WARCEF, Warfarin vs. Aspirin in Reduced Cardiac Ejection Fraction; HR, hazard ratio; CI, confidence interval; WATCH, Warfarin and Antiplatelet Therapy in Chronic Heart failure; COMMANDER HF, A Study to Assess the Effectiveness and Safety of Rivaroxaban in Reducing the Risk of Death, Myocardial Infarction, or Stroke in Participants with Heart Failure and Coronary Artery Disease Following an Episode of Decompensated Heart Failure; COMPASS, Cardiovascular Outcomes for People Using Anticoagulation Strategies; NAVIGATE-ESUS, New Approach Rivaroxaban Inhibition of Factor Xa in a Global Trial vs. Aspirin to Prevent Embolism in Embolic Stroke of Undetermined Source.

Our study is unique from these prior studies and randomized clinical trials, which largely evaluated the efficacy of antithrombotic therapy for *primary* prevention of stroke and cardiovascular events. Among patients with a recent stroke and HFrEF, the risk of recurrent stroke is considerably greater. In an exploratory subgroup analysis of the NAVIGATE-ESUS trial, including patients with a recent stroke and left ventricular dysfunction, investigators reported a 4.9% annualized risk of recurrent stroke/systemic embolism with antithrombotic therapy (12). Despite trial differences, this rate is numerically higher than that reported in WASH (18), WATCH (20), and HELAS (19), which included patients with low ejection fraction (~2% annualized risk of stroke/systemic embolism). Furthermore, in this subgroup analysis of NAVIGATE-ESUS, anticoagulation with rivaroxaban was associated with a significant reduction in the risk of recurrent stroke (HR 0.36, 95% CI: 0.14–0.93). Although there were numerically more major bleeding events with rivaroxaban vs. aspirin (5 vs. 0), there remained a significant treatment benefit with rivaroxaban for the secondary composite outcome of recurrent stroke, systemic embolism, myocardial infarction, or cardiovascular mortality (HR 0.51, 95% CI: 0.3–1.0). Given the advantage of rivaroxaban was driven by the higher risk of stroke/embolism in this population with a recent stroke, the risks of major bleeding are considered acceptable. In our study, we also found a similarly high risk of stroke recurrence as in NAVIGATE-ESUS (5.6% per year). However, we included patients treated with warfarin as well as any direct oral anticoagulant in the anticoagulant group. Therefore, we conducted a sensitivity analysis comparing treatment with any direct oral anticoagulant or low-molecular weight heparin against antiplatelet therapy, and still found no association between treatment and reduction in recurrent stroke risk. The lack of association of anticoagulation is likely driven by residual confounders (such as pre-morbid disability and unmeasured severity of comorbidities) and the short duration of follow-up.

In our analysis, large-vessel occlusion (LVO) was identified as a strong and independent predictor for recurrent stroke in patients with systolic heart failure and prior stroke, indicating that our retrospective cohort is a high-risk population. The Screening Technology and Outcome Project (STOP) Stroke Study was a prospective imaging-based study of stroke outcomes, which found that LVO was a strong predictor of mortality in patients. Furthermore, CHF was considered to be a strong risk factor in patients who were found to have an LVO when presenting with acute ischemic stroke (25). A recent retrospective cross-sectional study showed that a reduced left ventricular ejection fraction was more common among patients with ESUS after excluding patients with ipsilateral non-stenotic carotid atherosclerosis (26). Altogether, these findings support a growing idea that a subset of strokes classified as ESUS are likely to be cardioembolic in nature and thus may be responsive to differential antithrombotic regimen (12, 26).

### Limitations

Major limitations of the study include the small sample size with a short duration of follow-up and the high rate of death among patients in either treatment group. By comparison, it took a median of 10 months of follow-up from a nested cohort of 504 patients from NAVIGATE-ESUS to demonstrate a treatment effect with rivaroxaban over aspirin in patients with ventricular dysfunction (12). While PSM reduced the imbalance in the distribution of baseline characteristics, it left us with a small sample size. A larger sample using the entire cohort of eligible patients gave us the sufficient statistical power to detect a 20% difference in primary outcome rates in a multivariable model (based on some published data suggesting the benefit of anticoagulation in HFrEF) (3, 12). Our single-center experience, which involves the care of underserved patients with chronic and uncontrolled comorbidities, may also not be generalized to other centers and populations. Non-randomized treatment allocation is also a limitation and may be associated with unmeasured confounders. While the CHA_2_DS_2_-Vasc scores were similar between patient groups and this a useful indicator of bleeding risk (27), there may be other unmeasured differences that were used to individualize antithrombotic therapy. Lastly, there was an overrepresentation of male patients in the cohort, which may affect the generalizability of findings to female patients with ventricular dysfunction. However, the male-to-female ratio of ~3:1 is consistent with other trial data in patients with ventricular dysfunction (6, 12).

## Conclusion

In HFrEF patients with an acute stroke, there was no significant difference between antiplatelet and anticoagulant treatment in the prevention of recurrent stroke, major bleeding, or death. These data provide real-world support for prior randomized clinical trials, which have not consistently demonstrated the superiority of anticoagulation over antiplatelet therapy for HFrEF. The superiority of anticoagulation in this population would be best evaluated using a randomized clinical trial; however, there are no trials to the authors' knowledge that are currently planned for this target population. Several larger multi-center cohort studies, with hierarchical modeling of subgroups based on ejection fraction and other potential embolic sources in patients with cryptogenic infarction, are under way. Stratified analyses evaluating the risk of recurrent stroke, according to the degree of left ventricular dysfunction, comorbid conditions, and pre-existing disability, are important to identify which, if any, patients with systolic dysfunction and stroke may benefit from anticoagulation over antiplatelet therapy. Until better evidence is available, antithrombotic selection ought to be individualized based on the risk of recurrent stroke/systemic embolism and major bleeding complications (28). The most recent guidelines from the American Heart Association do not recommend anticoagulation to be used in place of antiplatelet therapy for patients with reduced ejection fraction, unless a myocardial infarction has also occurred (29).

## Data availability statement

The raw data supporting the conclusions of this article will be made available by the authors, without undue reservation.

## Ethics statement

The studies involving human participants were reviewed and approved by Cooper University Hospital Institutional Review Board (IRB). Written informed consent for participation was not required for this study in accordance with the national legislation and the institutional requirements.

## Author contributions

PP and JS were responsible for conceptualization, acquisition of the data, interpreting results, drafting of the manuscript, and revision of the manuscript for critical elements. JTi was responsible for data collection and interpreting results. JTh was responsible for manuscript revision and data interpretation. JS was responsible for statistical analysis of the data. JTi, AC, TS, SO, AG, SK, NV, AR, and TH were all responsible for data collection and review of manuscript for critical elements. PP and JS wrote the first draft of the manuscript. All authors commented on previous versions of the manuscript.
